# Adolescents’ Well-Being, Self-Esteem, and Academic Motivation as Bystanders: A Grounded Theory of Authenticity in Witnessing Bullying

**DOI:** 10.3390/bs15121656

**Published:** 2025-12-02

**Authors:** Thérèse Olsson, Robert Thornberg, Camilla Forsberg, Tomas Jungert

**Affiliations:** 1 Department of Psychology, Lund University, 221 00 Lund, Sweden; therese.olsson@psy.lu.se; 2 Department of Behavioural Sciences and Learning, Linköping University, 581 83 Linköping, Sweden; robert.thornberg@liu.se (R.T.); camilla.forsberg@liu.se (C.F.)

**Keywords:** school bullying, bystander, students’ perspectives, authenticity, self-esteem, study motivation

## Abstract

Bullying is increasingly understood as a group-based phenomenon in which bystanders play a critical role, yet little is known about how witnessing bullying affects bystanders’ self-esteem and academic motivation. The aim of this study was to explore adolescents’ perspectives on how witnessing bullying in school may be linked to their self-esteem and academic motivation. This qualitative study explored these experiences among 35 Swedish adolescents (22 girls and 13 boys, aged 14–16) using focus group interviews analyzed through constructivist grounded theory. The analysis generated a core category, Authenticity in witnessing bullying, reflecting how adolescents balanced empathic responses, self-image, and academic motivation when confronted with bullying. In addition, six interrelated categories emerged: (1) sympathetic wounding, (2) relationship buffering, (3) fear-driven avoidance, (4) academic shielding, (5) academic draining, and (6) normalization of bullying. Witnessing bullying affected participants’ feelings of authenticity, self-esteem, coping strategies, and academic focus. Academic motivation was shaped divergently: some students withdrew and lost motivation, while others increased focus on grades to secure transfer to a safer school environment. The theory offers a framework that is grounded in participants’ accounts that helps the understanding of how authenticity shapes the social context, emotional experience, and academic engagement. Interventions that address the emotional and motivational consequences of witnessing bullying and foster supportive school climates, empowering students to act constructively, are needed.

## 1. Introduction

Bullying is increasingly recognized as a complex relational phenomenon that extends beyond dyadic aggressor–victim dynamics, involving broader peer group processes and social contexts (Rodkin et al., 2015; Salmivalli & Peets, 2018). Within these dynamics, bystanders—those who witness bullying—play a significant role in either reinforcing or mitigating such behavior. Research indicates that bystanders frequently remain passive or even support the aggressor rather than intervene on behalf of the victim (Salmivalli et al., 2011). This reluctance to help may be shaped by fear of retaliation, social hierarchies, peer pressures, or uncertainty about how to respond (Forsberg et al., 2014; Kubiszewski et al., 2019; Spadafora et al., 2020; Strindberg et al., 2020; Williams et al., 2018).

Previous research has identified a range of psychological and contextual factors that influence bystander behavior, including empathy, empathic anger, moral sensitivity, moral disengagement, self-efficacy, attitudes, peer norms, and peer status (Jungert & Perrin, 2019; LaChance et al., 2025; Lambe & Craig, 2023; Pozzoli et al., 2017; Xie et al., 2023; Yang & Gao, 2023). However, research has predominantly relied on quantitative methods, such as surveys and psychometric assessments (e.g., Nickerson et al., 2014; Sijtsema et al., 2015), which may limit the understanding of how children interpret and make sense of their experiences as bystanders.

Qualitative approaches offer an alternative perspective by exploring the lived experiences and meaning-making processes of those who witness bullying. This methodological approach can provide deeper insights into how children articulate their experiences, explain their behavioral motives and responses, and attribute meanings to their roles in bullying situations (Patton et al., 2017). Nonetheless, a relatively small number of qualitative studies have investigated children’s and adolescents’ own perspectives on bystanding. Prior work has primarily focused on bystanders’ motivation, reasoning, and actions (Forsberg et al., 2014, 2018; Spadafora et al., 2020; Strindberg et al., 2020; Thornberg et al., 2018; Williams et al., 2018). For example, Forsberg et al. (2018) identified five broad factors shaping students’ reasoning as bystanders: informed awareness, bystander expectations, personal feelings, behavioral seriousness, and sense of responsibility—highlighting the situational complexity involved in bystander decision-making.

Importantly, while previous research has paid substantial attention to bystanders’ motivations and actions, there remains a notable gap concerning the potential emotional and psychological impact on those who witness bullying. Specifically, limited knowledge exists about how being a witness may affect students’ self-esteem and academic motivation, two key indicators of academic and emotional well-being (Acosta-Gonzaga, 2023; Holzer et al., 2022). Given the pivotal role of social relationships in shaping children’s school experiences and development (O’Moore & Minton, 2005), it is crucial to explore how occupying the role of bystander may influence their self-concept and school engagement.

This study addresses this gap by examining how young adolescents who have witnessed bullying narrate their experiences in focus group interviews. Employing a constructivist grounded theory approach (Charmaz, 2014), the study seeks to develop a nuanced understanding of how students make sense of being bystanders and how these experiences may influence their self-esteem and academic motivation. The goal is to contribute to and conceptualize an emic perspective on the social and emotional consequences of witnessing bullying, where an emic perspective refers to understanding of the phenomenon from those who experience it firsthand—the insiders.

### 1.1. Self-Esteem

Self-esteem can be understood as an individual’s overall sense of self-worth and perceived competence across different domains of life, forming a central component of psychological well-being (von Soest et al., 2016). For school-aged children, healthy self-esteem is closely tied to mental health, resilience, and social adjustment (Moksnes & Reidunsdatter, 2019). While much research has examined the relationship between self-esteem and direct involvement in bullying as a perpetrator, victim, or defender (Tian et al., 2025; Tsaousis, 2016), less is known about how witnessing bullying might influence adolescents’ feelings and evaluation of their self.

Previous studies have demonstrated that self-esteem may vary across different roles in bullying situations. For example, Salmivalli et al. (1999) found that students with higher self-esteem were more likely to defend victims, while those with lower self-esteem might seek social standing through aggressive behaviors (Gini, 2006; Juvonen & Galván, 2008). However, such patterns do not fully capture the nuanced and potentially fluctuating ways in which bystanders who witness, but do not directly participate in, bullying interpret these events and position themselves within them. Recent work by Jungert et al. (2025) suggests that witnessing bullying may influence students’ perceptions of themselves, their peers, and their classroom environment, underscoring the social and emotional impact of these experiences. Studies have also shown that witnessing bullying at school or online is associated with mental health problems (Doumas & Midgett, 2020; Midgett & Doumas, 2019; Rivers et al., 2009; Wright & Wachs, 2023). Thus, being a passive bystander may evoke a complex mix of emotions, moral evaluations, and reflections on one’s own social standing, all of which can affect the broader self-esteem processes.

From a constructivist grounded theory perspective (Charmaz, 2014), exploring how adolescents make sense of witnessing bullying provides an opportunity to understand self-esteem not as a static trait, but as an evolving, socially embedded process. In this view, self-esteem is shaped through ongoing interactions, meanings, and negotiations within the school environment. Accordingly, this study seeks to generate a conceptual understanding of how 15-year-old students perceive and interpret their role as witnesses to bullying, and how these experiences may influence their sense of self-esteem and academic motivation.

By adopting a qualitative approach, the aim is to capture the richness of students’ subjective accounts.

### 1.2. Motivation

Self-Determination Theory (SDT; R. M. Ryan & Deci, 2017) conceptualizes academic motivation as a continuum ranging from highly self-determined to more controlled forms. At one end of this continuum lie autonomous motivations, such as intrinsic motivation (engaging in learning for enjoyment or interest) and identified regulation, in which students pursue schoolwork because they personally value its importance. At the opposite end are controlled forms of motivation, such as introjected regulation, driven by internal pressures like guilt, and external regulation, where behavior is governed by external rewards or punishments. Amotivation represents a lack of intention or drive to engage in learning activities at all.

Research has consistently linked autonomous motivation to positive outcomes, including self-esteem, persistence, and academic achievement, whereas controlled motivation is often associated with less desirable outcomes, such as anxiety and disengagement (Guay et al., 2010). However, much of this evidence is variable-centered and overlooks how different forms of motivation may coexist and interact within students’ lived experiences.

From a constructivist grounded theory perspective, motivation is not only a set of measurable constructs but also a lived and interpreted phenomenon shaped by students’ interactions with others and the school environment (Keane, 2024; Lee et al., 2018). Witnessing bullying may influence students’ motivation in subtle ways, for instance by altering their sense of belonging, perceived competence, or belief in the value of schooling. Through a qualitative research approach, this study examines how bystanders make sense of their motivation to engage with school in contexts where the social climate is disrupted by bullying, and how this process intersects with their evolving self-esteem.

In line with SDT, adolescents’ motivation is closely intertwined with their broader sense of self and perceptions of the school climate. Recent research indicates that self-esteem is connected to students’ engagement with learning—often indirectly through beliefs about competence and task value (Zhao et al., 2021). Evidence also suggests that bystander status may be linked to lower self-esteem, implying that merely witnessing aggression can unsettle students’ sense of worth and belonging, with potential implications for their willingness to participate, persist, and take on academic challenges (González Moreno & Molero Jurado, 2024). Large-scale syntheses further demonstrate that more self-determined forms of motivation are consistently associated with higher levels of well-being, engagement, and achievement, and that need-supportive contexts (those that nurture autonomy, competence, relatedness) help cultivate such motivation (Bureau et al., 2022). Seminal longitudinal work underscores the unique role of intrinsic motivation in predicting academic outcomes over time (Taylor et al., 2014). Recent findings on witnessing school bullying further emphasize that bystanders’ perceptions, roles, and self-evaluations are implicated when aggression occurs, highlighting the need to theorize motivation and self-esteem as co-constructed within social contexts; precisely the kind of process a constructivist GT study can elucidate (Jungert et al., 2025).

This study also draws upon social psychological and developmental theories, including symbolic interactionism (Blumer, 1969; Charon, 2010; Hewitt & Shulman, 2011), social cognitive theory (Bandura, 2014), and models of bystander decision-making (Dovidio et al., 2017; Latané & Darley, 1970). Together, these frameworks emphasize that children’s behavioral responses are shaped not only by the bullying event itself but also by their interpretations of the social situation, in other words, the opportunities and constraints they perceive and the lines of action they are involved in, as well as deem feasible (Närvänen & Näsman, 2007).

### 1.3. Authenticity

Authenticity, which can be defined as “the sense or feeling that one is currently aligned with one’s true or genuine self, that one is being his or her real self” (Sedikides et al., 2017, p. 521), is increasingly recognized as central to adolescents’ psychological functioning. In contrast, inauthenticity, or the sense of acting as a false self, has been linked to maladaptive outcomes such as lower self-esteem and diminished well-being (Kernis & Goldman, 2006; R. M. Ryan & Deci, 2017). Within Self-Determination Theory, authenticity is closely tied to autonomy: individuals feel authentic when their actions align with personal values and are experienced as self-authored, and inauthentic when they act primarily to gain acceptance or avoid rejection (W. S. Ryan & Ryan, 2019). Importantly, authenticity emerges in specific contexts and situations, depending on whether basic psychological needs for autonomy, competence, and relatedness are supported or thwarted (R. M. Ryan & Deci, 2017).

During adolescence, identity formation involves negotiating multiple social roles and expectations. The degree to which these identities become integrated predicts well-being, self-esteem, and resilience (Peets & Hodges, 2018; Sutton, 2020). Experiences of authenticity are fostered by supportive environments and peer relationships (Gueta & Berkovich, 2022), whereas need frustration or self-silencing in relational contexts can undermine authenticity (Alchin et al., 2024; Jack & Dill, 1992). For students who witness bullying, authenticity may be challenged if they feel pressured to act against their values, remain silent, or suppress their true responses. In this sense, authenticity provides a useful conceptual lens for understanding how witnessing bullying can shape both self-esteem and motivation in school.

### 1.4. The Current Study

Bullying is increasingly recognized as a group-based phenomenon in which bystanders play a critical role. However, limited research exists on the impact that witnessing bullying exerts on adolescents’ self-esteem and academic motivation, two key indicators of psychological and educational well-being. The aim of the current study was, therefore, to explore adolescents’ perspectives on how witnessing bullying in school may be linked to their self-esteem and academic motivation. This qualitative study involved 35 Swedish adolescents from five schools, utilizing constructivist grounded theory to explore their perspectives on and experiences of witnessing school bullying.

## 2. Materials and Methods

### 2.1. Participants

A purposeful sampling procedure was employed to include schools with variations in socioeconomic context. The dataset was obtained from five middle schools located in both large urban areas and smaller municipalities in Sweden, representing a range of socioeconomic and academic environments. These schools varied moderately in academic achievement and size (approximately 400–700 students). Institutional consent was obtained from principals and schoolteachers, and students were invited to participate through both written and verbally informed assent in focus groups.

In total, thirty-five students participated in the study (22 girls and 13 boys; aged 14 to 16 years), all of whom were enrolled in either eighth or ninth grade in middle school. Four students were selected to participate in individual interviews, while the remaining students took part in focus groups comprising approximately four participants each (three to four students). Both types of interviews followed the same semi-structured guide. During analysis, we compared responses from individual interviews and focus groups and found no meaningful differences in the patterns or themes reported. As a result, the findings presented in Section 3 integrate data from both sources without separate reporting.

All participants were assured confidentiality of all information provided, and the transcribed interviews were anonymized prior to analysis. The research was conducted in accordance with the Ethical Principles of Psychologists and Code of Conduct (APA).

### 2.2. Data Collection and Procedure

To examine students’ experiences as bystanders to bullying, their self-esteem, and academic motivation, we developed semi-structured interview guides.

Before the focus group discussions began, participants were informed that the study explored adolescents’ general experiences of witnessing different types of school bullying, including both their own experiences and what they had observed others experiencing. The moderator introduced the purpose of the study, the confidentiality procedures, and the ground rules for respectful and anonymous discussion. Participants were informed that the conversation would address how identification with school/class, relationships with teachers, and the type of bullying witnessed may influence their reactions and roles in bullying situations, as well as how witnessing bullying could affect their own needs (frustration, self-esteem, and study motivation). The interview guide included open-ended questions on these themes, with follow-up probes added as needed in accordance with the iterative nature of grounded theory methodology.

Two trained research assistants conducted the interviews in private rooms at each school. Their training, delivered by two researchers with expertise in qualitative methods and interview techniques, focused on both the proper use of the interview guides and adherence to study procedures. Consistent with the emic approach, the use of semi-structured interviews enabled participants to assume the role of experts of themselves and their everyday social life in school, freely expressing their thoughts, emotions, and personal experiences (Patton et al., 2017), while the interviewers adopted the stance of interested learners (Charmaz, 2014). Interviews lasted approximately 25 to 60 min.

At the start of the discussion, a brief definition of school bullying was provided to ensure a shared understanding among participants. Students were also asked to describe and identify behaviors they considered to be bullying during the focus groups. During the interviews, interviewers clarified and elaborated on questions when necessary, either for follow-up on participants’ responses or to accommodate the ongoing analytic process. This iterative approach to data gathering and analysis, including the later theoretical sampling process, occasionally prompted additions of new themes and questions to the interview guide.

Participants were asked to share their current views of themselves and others in the role of bystander, as well as to recount their experiences of witnessing bullying. They described personal thoughts and emotional responses to observed bullying situations and considered how these experiences related to their own self-esteem and motivation for academic engagement.

To reduce the risk of social desirability bias, the interviewers listened attentively, posed follow-up questions, and maintained a non-judgmental stance toward all perspectives presented (Mandell, 1991; Mayall, 2000). Interviews were conducted in Swedish, audio-recorded, and transcribed verbatim.

### 2.3. Data Analysis

Data were coded following the constructivist grounded theory approach, which incorporated initial, focused, and theoretical coding (Charmaz, 2014; Charmaz et al., 2018). A constructivist position was adopted to explore participants’ understandings of bullying motives, grounded in the view that both data and theory are co-constructed through the interactions between researchers and participants (Charmaz, 2014). This epistemological stance fostered ongoing reflexivity throughout the research process (Charmaz, 2014; Charmaz et al., 2018).

Consistent with the informed grounded theory’s emphasis on theoretical agnosticism and theoretical pluralism, the analysis maintained openness and sensitivity to the studied field, the participants, and the data (Thornberg, 2012). Self-determination theory, social cognitive theory, symbolic interactionism, models of bystander decision-making, and Bronfenbrenner’s (1977, 2000) ecological systems theory served as a loose and provisional conceptual framework and a starting point for the study. Pre-existing theories and concepts were regarded as provisional, fallible, and partial (Themelis et al., 2023; Thornberg, 2012) and functioned as sensitizing concepts (Blumer, 1969), providing a broad conceptual orientation without determining the analytic process (Charmaz, 2014; Thornberg, 2012).

Data analysis began with initial coding, employing constant comparison across data segments, codes, and categories to discern and conceptualize salient actions and meanings. Initial codes were constructed through close line-by-line coding of the data. Subsequently, the analysis advanced to focused coding, where the most significant or frequent initial codes were identified as focused codes and, therefore, examined in greater depth, forming the foundation for category development. The iterative development of categories involved theoretical sampling, such as adding new questions in subsequent interviews to elaborate and refine emerging categories. Focused coding facilitated the identification of a core category, defined as the most significant and frequently represented category in the data (Glaser, 1998).

Theoretical coding was conducted in parallel with focused coding, exploring interrelationships among categories to support the development of a grounded theory (Glaser, 1978, 1998). Throughout the analytic process, memo-writing was employed to document analytical insights, reflections, and theoretical linkages. Comparison of data, codes, and categories with extant literature, the preliminary conceptual framework, and previous research—supplemented by memos on similarities, differences, and potential connections between developed concepts and the literature—was an integral part of the analytic strategy. During theoretical coding, categories were examined in relation to one another and interpreted using sensitizing concepts from Self-Determination Theory (Deci & Ryan, 1985; R. M. Ryan & Deci, 2017), an ecological perspective (Bronfenbrenner, 1977, 2000), and the psychological construct of self-esteem to provide additional and provisional interpretative lenses. The core category, Authenticity in witnessing bullying, was further developed by exploring its connections to self-esteem, coping strategies, and academic motivation. Adolescents’ experiences of authenticity—defined as alignment with their values and “real me”—shaped their responses to bullying, with some participants increasing their academic focus and others withdrawing or avoiding, reflecting differences in perceived autonomy, competence, and relatedness. Memo-writing and constant comparison guided this analytic process, ensuring that pre-existing theories could be informed, but not constrain, the analysis, maintaining groundedness (Charmaz & Thornberg, 2021), in other words, making sure that our constructed grounded theory would be grounded in the data.

Upon completion of coding, emergent concepts were constructed from the data, serving as the basis for the core category and its associated categories, as presented in the Section 3. This analytic approach enabled the integration of participants’ lived experiences with relevant theoretical perspectives from the literature while staying grounded in the data.

## 3. Results

The analysis of participants’ accounts of witnessing school bullying revealed distinct patterns related to protective relationships and empathetic responses toward victims. Another salient pattern pertained to the impact on participants’ well-being, encompassing avoidance strategies, heightened academic focus, coping mechanisms, and the development of a fragile self-image and diminished self-esteem. These patterns were shaped by the participants’ relationships with both perpetrators and victims. Notably, a recurrent social process emerged that constituted the core category: Authenticity in witnessing bullying. This core category encapsulated how adolescents’ sense of being their “real selves” shaped both their emotional responses and academic orientation when confronted with bullying.

Adolescents who expressed an ability to act authentically, for instance by showing empathy, offering help, and maintaining a coherent sense of self, reported that they created “a space of their own” where they felt heard without having to defend or justify themselves. As one participant noted, “I can stand up for myself. For those who can’t, I try to help.” Another student described a similar sense of groundedness in their own identity and relationships, emphasizing that they gravitated toward peers they genuinely liked rather than conforming to broader peer pressures: “You usually find the people you like yourself or share interests with, and then you become better friends with them. It’s not that people dislike each other; it’s just that people prefer spending time with those they know in their own groups, if you can call it that.”

A different participant illustrated authentic action even when it involved confronting a friend, describing how intervening ultimately strengthened both their self-esteem and the friendship itself: “In my previous school, a boy was being bullied because of his appearance. One of my friends was the one bullying him, so I spoke up against it. We ended up arguing, and another friend went to get a teacher. Afterwards, we talked about it, and he stopped. We’re still very good friends today. It strengthened my self-esteem and motivation—I felt important, and we supported each other more after that.”

Such authenticity was associated with higher self-esteem and autonomous academic motivation. Conversely, others constrained their authentic selves out of fear of negative reactions. As one participant noted, “I’ve become more afraid to express myself when I hear how people get attacked for speaking up. To fit in, you’d rather do nothing. So, I’ve become more cautious about saying what I think at school or in other open settings.” Another student described a similar tension when witnessing bullying by their own friends, highlighting how social loyalty restricted authentic action: “But at the same time, I thought, you know, they were my friends. They were also the ones bullying him. And I—I can’t speak up. They’re my friends; I can’t say anything. I barely know the guy. I know my friends much better, so it became like—I couldn’t say anything. That’s how it was.” When asked whether they intervened, the student added: “No, I didn’t do anything in that situation.”

Others described how a hostile or negative classroom climate reduced their ability to remain authentic altogether: “When you see that kind of thing and experience that kind of atmosphere, it becomes a factor that makes you want to stay home. Like, ‘Damn, I don’t want to go to school today.’ It’s just a bad environment and a bad atmosphere.”

These adolescents typically described diminished self-esteem and reduced academic motivation, reflecting constraints in autonomy and belonging.

Academic motivation appeared to diverge into two distinct, but theoretically coherent pathways. For some, witnessing bullying undermined self-image and self-esteem and created a heightened fear of social exposure, leading to avoidance-oriented strategies aimed at remaining unnoticed, decreased class attendance, and reduced academic engagement. For others, witnessing bullying activated a more approach-oriented coping response that increased their extrinsic academic motivation: attaining higher grades was perceived as a way to gain entry to better upper secondary schools and thereby avoid exposure to the same bullies. These contrasting responses suggest that perceptions of bullying, self-esteem, and academic motivation interact in complex and dynamic ways. Rather than producing uniform effects, witnessing bullying appears to elicit different motivational trajectories depending on the coping strategies students adopt.

(1) Sympathetic wounding;
(2) Relationship buffering;
(3) Fear-driven avoidance;
(4) Academic shielding;
(5) Academic draining;
(6) Normalization of bullying.

The relationships among these six categories provide a grounded understanding of the complexity underpinning the research questions. Each category illustrates dimensions of adolescents’ experiences, and together they reveal broader theoretical patterns related to the core category, Authenticity in witnessing bullying. In this way, the categories collectively contribute to middle-range theoretical insights, showing how self-esteem, academic motivation, and experiences of bullying are intertwined, without implying that each category represents an independent grounded theory.

### 3.1. Sympathetic Wounding

Sympathetic wounding refers to the emotional process whereby students experience feelings of sadness, compassion, or vicarious hurt when witnessing others become targets of bullying. Participants emphasized that witnessing bullying had a pronounced emotional impact. They expressed sympathy for victims, describing feelings of sadness or compassion when observing peers being targeted. For several students, the emotional response was accompanied by a sense of moral obligation or responsibility, even when they did not intervene directly. Rather than confronting perpetrators, some students opted to comfort the victim or demonstrate what we termed a sympathetic presence, meaning that through their presence, they sought to express sympathy and support for the victim. As one participant put it, “We didn’t do anything against those who were mean, but we showed that we were there… We comforted, and they listened.” Others reflected on how witnessing the suffering of friends personally affected them, exemplified by remarks such as, “It affects you to know that your friend is being exposed,” and “I really just felt sorry for her.” Nonetheless, not all participants reported experiencing sympathetic concern. In some cases, the nature or quality of the relationship with the victim modulated their response: “With him, I don’t really care, because he is not a nice person, at least not toward me, so therefore I don’t care if someone says something mean to him.” This illustrates that empathy and authenticity were not universally elicited but were instead contingent on relational dynamics.

### 3.2. Relationship Buffering

In addition to sympathetic concern, participants underscored the significance of protective and supportive relationships. Relationship buffering refers to the process by which supportive interactions and emotionally available relationships with significant others mitigate or reduce the negative or distressful emotional consequences of witnessing bullying by providing stability, reassurance, and affirmation. Several described how offering emotional or practical help, or simply being available to talk, could make a meaningful impact on victims. For example, one student recalled, “After we [the participant and her friend] had talked with a friend for a while, it got better.” Others emphasized how appreciation from the victim reinforced their own sense of having contributed positively: “It felt good, because she said afterwards that she appreciated it.” Such experiences often enhanced participants’ sense of authenticity and bolstered their self-esteem. Supportive relationships were also linked to the role of teachers, who could provide stability and reassurance. As one participant explained, “There are some teachers who I think people listen to more… a combination of being kind and strict is good.” These narratives illustrate how both peer and teacher relationships could buffer the negative effects of bullying, foster authenticity, and encourage students to remain engaged in school. Thus, relationship buffering operates through concrete actions, emotional support, or simply being present, fostering authenticity and strengthening self-esteem in students witnessing bullying.

### 3.3. Fear-Driven Avoidance

In addition to expressions of concern and support, many participants described how fear distinctly influenced their experiences of witnessing bullying. Fear-driven avoidance refers here to behavioral strategies motivated by anticipatory fear and enacted to minimize the perceived risk of being attacked or harmed by bullies, typically by withdrawing from specific environments, activities, or social interactions associated with bullying. The desire to remain unnoticed by bullies—since being seen could increase their vulnerability to becoming targets themselves—was occasionally articulated as a strategy. Students sometimes avoided specific places, classes, or peers associated with bullying. As one participant explained, “I didn’t want them to see me… I didn’t dare go to the gym.” Others described feeling tense in corridors or classrooms, for example: “It was a bit tough to go to school because I didn’t really know what would happen,” and “I didn’t dare walk past them. There was a place in the corridor where it happened the most.”

This fear was also intertwined with a sense of vulnerability arising from identification with the victim. As one participant reflected, “That person is like me—if it could happen to him, it could happen to me. Then you start wondering if you even want to go to school.” Such insights highlight how witnessing bullying not only prompted situational avoidance but also, in some cases, led to considerations of disengaging from school altogether.

Fear also constrained participants’ willingness to intervene on behalf of victims, even when they felt sympathetic wounding. As one student reflected, “And I didn’t do anything either. Because… it feels like I can’t say anything without being subjected to comments and stuff.” This illustrates how fear of becoming a target or being judged prevented students from acting, reinforcing the sense of vulnerability and contributing to avoidance behaviors.

Several participants also explained that they avoided group work with peers involved in bullying or attempted to keep a low profile during lessons: “I wanted the lesson to be over,” and “I focused on avoiding them.” These strategies often contributed to diminished self-esteem, a fragile sense of self, and, in some cases, reduced autonomous motivation to attend school: “I haven’t wanted to be in school.” In this way, avoidance emerged as a means of coping with fear, but at the expense of both authenticity and academic engagement.

A more covert form of fear-driven avoidance is self-silencing, which refers to being silent and blending in with peers when witnessing bullying due to anticipatory fear of being attacked or harmed by bullies. Many students described remaining silent or attempting to blend in, even when they experienced strong empathy for victims. This passivity was often attributed to fear of social repercussions and was experienced as a constraint on authenticity: “Because I just kind of have to act along as everyone else, which I don’t really like having to… That’s why I prefer home a lot. I can be more myself.”

### 3.4. Academic Shielding

Academic shielding refers to when academic striving serves as a mechanism to cope with witnessing bullying or avoid the hostile peer climate in general, providing either psychological safety, an escape route, or a means of maintaining self-esteem and authenticity when direct social engagement feels risky. Several participants described channeling their energy into schoolwork as a means of dealing with school bullying and unsafety. For these students, academic performance functioned both as an avoidance strategy and as a path to escape, with grades regarded as a ticket to safer educational environments in the future: “If I just focus and do the least I can… then I will get further in life.” Others noted that witnessing bullying heightened their determination to succeed in school: “I focused even better in school,” and “I got a lot of motivation.”

This extrinsically regulated form of academic motivation often coexisted with efforts to maintain self-esteem, as students emphasized striving to remain relaxed or authentic in the school setting. The pursuit of academic achievement provided structure and a sense of progress, even as peer interactions sometimes inhibited their ability to be themselves.

### 3.5. Academic Draining

Academic draining refers to the depletion of cognitive and emotional resources that results from persistent distractions, interpersonal conflicts, and a hostile or conflict-laden school environment related to repeatedly witnessing bullying. Participants described how bullying dynamics created significant distractions that undermined their ability to concentrate on schoolwork. The presence of bullying, aggression, and peer conflicts in school absorbed attention and left limited cognitive and emotional capacity for academic focus. As one student explained, “You don’t feel as motivated to work… there’s more focus on the other stuff, so sitting there and studying feels almost impossible when you see everything happening around you.”

Others emphasized the persistent concern with peer loyalties: “Yes, you have to be on this side, no, you have to be on that side. And that has affected me so that I think about it instead of studies.” For some, uncertainty regarding the stability of friendships overshadowed their school tasks: “Will I have any friends left this week? That takes up more energy than school.” In other instances, students did not necessarily experience a direct loss of academic motivation themselves but nonetheless perceived the school climate as deteriorating: “No, I don’t think it affects whether I feel motivated or not. But of course, it creates a worse general environment at school.” These accounts illustrate how the peer dynamics and social processes both within and surrounding bullying events could shape the learning environment and impede academic engagement, even when individual motivation to succeed academically remains intact.

### 3.6. Normalization of Bullying

Normalization of bullying, in the context of school bullying, refers to the process by which repeated exposure to bullying leads individuals to perceive such behavior as a regular, inevitable, and accepted part of school life. “I just thought it was a normal thing that happens in school,” and “I think it happens at every school, really.” Through continual witnessing of bullying, some students described how they became desensitized and emotionally detached, seeing bullying as “normal” or “everyday noise,” as one of the participants put it, “You almost block it out in a way so that it becomes… everyday noise by now.”

While normalization of bullying functioned as a coping mechanism, it simultaneously reflected a weakening of authentic emotional and behavioral engagement as students distanced themselves from their genuine affective responses. Within this category, emotional distancing and moral resignation were understood as consequences of the normalization process itself, emerging as students repeatedly encountered bullying and came to view it as routine and unremarkable. One participant articulated the sense of exhaustion that emerged from continuous exposure: “Because it happens so often that I couldn’t be bothered to, you know, do anything. It’s like, if something happens way too many times, eventually you just stop caring.”

Some students expressed this emotional distancing even more explicitly by noting that bullying did not affect them personally: “It doesn’t really affect me, because it’s not directed at me.” Such statements highlight how self-protection was achieved through disengagement, even when the harm inflicted upon peers was clearly recognized. The normalization of bullying and its accompanying emotional distancing resulted in a sense of moral resignation, meaning that they felt too emotionally detached and indifferent to become morally engaged.

Although the study was conducted in Sweden, the patterns identified here align with findings from cross-national research showing that adolescents’ responses to bullying are shaped primarily by peer-group dynamics rather than by country-specific cultural norms. Large comparative studies demonstrate that structural similarities in bullying and bystander behavior are common across diverse educational systems, even when the prevalence of bullying varies or when linguistic terms differ (Smith, 2016). Research on bystanders in multiple countries likewise points to comparable social mechanisms, such as fear of social sanctions, concerns about belonging, and moral tension when witnessing aggression (Kärnä et al., 2013). These consistencies suggest that while the Swedish school context provides the immediate setting for the present study, the core social processes described below—particularly those related to authenticity, relational safety, and peer-group dynamics—are likely to be relevant in other cultural and educational contexts as well.

Notably, even in school contexts described as generally positive, experiences of other peers’ exclusion and subtle forms of marginalization remained present: “There is always someone who is kind of outside in some way.” In some cases, normalization of bullying is manifested through explanations that position bullying as a response to perceived differences or deviations among certain peers. Such reasoning effectively displaced responsibility from the act of bullying itself onto the victim. By framing bullying and peer exclusion in these terms, students not only blamed the victim but created social distance from the targeted peer and affirmed their own sense of belonging, thereby diminishing the likelihood of empathetic and genuine involvement or intervention.

Normalization of bullying also carries important moral implications. As Gutzwiller-Helfenfinger and Perren (2022) argue, moral meaning-making in schools is shaped by social-ecological processes through which harmful behaviors come to be seen as ordinary or socially acceptable. In our study, repeated exposure not only fostered moral desensitization but also reduced adolescents’ perceived responsibility to act, narrowing the space for authentic moral agency. As bullying becomes normalized within the peer climate, the boundaries of what counts as “wrong” or “intervenable” erode, undermining adolescents’ autonomy and relatedness both emotionally and morally.

### 3.7. A Grounded Theory of Authenticity in Witnessing Bullying

Becoming a bystander to bullying in school can evoke discomfort and distress. While it may prompt a sense of moral obligation to intervene or speak up, feelings of fear, inadequacy, and uncertainty can hold students back and lead to continued passivity (Forsberg et al., 2018). Repeated exposure to bullying at school may also contribute to experiences of school unsafety. In this study, we found that students who witness bullying struggle with the desire to remain true to themselves—a challenge that bullying situations often provoke. The analysis of the interview data resulted in a grounded theory of authenticity in witnessing bullying, which concerns how students, in various ways, strive to maintain their authenticity (being true to themselves and others) when they observe bullying at school.

Together, the six categories illustrate the complex ways in which adolescents navigate their experiences of witnessing bullying, each interacting with the core category, Authenticity in witnessing bullying. Sympathetic wounding and relationship buffering generally support authenticity linked to defending when witnessing bullying, whereas fear-driven avoidance, academic draining, and normalization of bullying constrain authentic expression and may diminish defending. Academic shielding can serve both to protect authenticity (being a good student in academic terms) and as a coping strategy, because redirecting attention from perceived moral failure and the inability to assist the victim toward academic pursuits can help maintain a positive self-image and a sense of authenticity.

Importantly, these categories do not operate in isolation but form a dynamic, process-oriented model in which adolescents negotiate the tension between authenticity and self-protection. Their strategies for maintaining or constraining authenticity are shaped by contextual conditions such as relational safety, perceived social risks, and peer climate. When the environment supports openness and a sense of belonging, authenticity is sustained through empathy and engagement. Conversely, when fear or hostility prevails, adolescents rely on avoidance, normalization of peer victimization, or emotional detachment to cope, thereby protecting themselves but compromising authenticity, linked to moral agency, self-esteem, and autonomous academic motivation.

Together, these processes constitute a grounded theory of authenticity in witnessing bullying—a social psychological process through which adolescents manage threats to self and relatedness while striving for safety and agency in bullying contexts. The model underscores how relational support and peer climate influence the strategies adolescents adopt, and how these strategies in turn affect their motivational needs for autonomy, relatedness, and competence (R. M. Ryan & Deci, 2017). Thus, the theory provides a framework grounded in participants’ accounts for understanding of how adolescents’ concern with maintaining authenticity shapes social context, emotional experience, and academic engagement.

## 4. Discussion

The aim of this study was to explore adolescents’ perspectives on how witnessing bullying in school may be associated with their self-esteem and academic motivation. Drawing on adolescents’ experiences and viewpoints, we developed a grounded theory comprising a core category—a basic psychological process that we labeled ‘authenticity in witnessing bullying’. This core process refers to how students strive to remain true to themselves and others when observing bullying. This process encompasses six dimensions (sympathetic wounding, relationship buffering, fear-driven avoidance, academic shielding, academic draining, and normalization of bullying) and is connected to emotional responses, self-perception, and motivation. Adolescents who were able to act authentically by expressing empathy, offering help, and maintaining a coherent sense of self tended to sustain higher self-esteem and autonomous academic motivation. This finding aligns with previous research linking authenticity to both self-esteem (Kernis & Goldman, 2006) and self-determined motivation (R. M. Ryan & Deci, 2017), while extending earlier work by illustrating how authenticity is experienced and negotiated in a real school context characterized by bullying.

Together, the six interrelated categories illustrate the complex ways in which adolescents navigate the experience of witnessing bullying, each interacting with the core process of authenticity in witnessing bullying. Sympathetic wounding and relationship buffering generally supported authenticity by fostering empathy, self-esteem, and academic engagement. In contrast, fear-driven avoidance, academic draining, and normalization of bullying constrained authentic expression and often diminished intrinsic or autonomous motivation. Academic shielding occupied a more ambivalent position, serving both to protect authenticity by enabling emotional distance to function as an avoidance strategy that limits engagement. Collectively, these categories depict a dynamic interplay between relational support, personal coping strategies, and the broader peer climate that shapes adolescents’ well-being and academic motivation. The findings propose a conceptual yet provisional framework—grounded in adolescents’ reported experiences—for understanding how authenticity mediates the emotional and academic consequences of witnessing bullying.

From a self-determination theory perspective (R. M. Ryan & Deci, 2017), these findings can be understood through the dynamics of need satisfaction and frustration. When students were unable to act authentically, due to fear, peer pressure, or moral disengagement, their need for autonomy was undermined. As W. S. Ryan and Ryan (2019) emphasize, authenticity entails congruence between inner experience and outward expression. When this congruence is constrained, adolescents may experience a loss of volition and internal coherence. Witnessing bullying also impeded the satisfaction of the need for relatedness. Observing peers being subjected to exclusion, hostility, or peer aggression often evoked feelings of insecurity and disconnection, thereby inhibiting empathic concern and genuine social engagement. Such relational strain sometimes prompted self-protective coping strategies (Parris et al., 2020), which, in turn, weakened their sense of belonging.

The need for competence was likewise affected, both directly and indirectly. Classrooms in which ridicule or derogatory commentary toward victims occurred created atmospheres of evaluative threat, undermining students’ need for competence even when they were bystanders. This aligns with prior findings showing that bystanders are less inclined to defend victims in morally disengaged classroom climates (Sjögren et al., 2021) and where antibullying norms are weaker (Garandeau et al., 2022; Thornberg et al., 2023). Bullying prevalence also tends to be higher in classroom peer contexts where students frequently laugh at and encourage perpetrators during incidents (Kärnä et al., 2010; Nocentini et al., 2013; Salmivalli et al., 2011). When coping strategies involved avoiding certain lessons or peers, opportunities for accomplishment naturally diminished, further reinforcing a sense of incompetence. Thus, constrained authenticity not only restricts self-expression but also erodes academic motivation.

We then interpret these patterns through the lens of self-determination theory (R. M. Ryan & Deci, 2017), which illustrates how relatedness fostered emotional safety, autonomy was preserved through genuine self-expression, and competence was strengthened through sustained engagement and academic participation. These results underscore that authenticity and motivation are profoundly relational and contextually situated processes rather than fixed individual traits. Based on our data, adolescents who engaged in what we term relationship buffering reported feeling safe to express empathy and sustain their sense of self. We then interpret these patterns through the lens of self-determination theory (R. M. Ryan & Deci, 2017), which illustrates how relatedness fostered emotional safety, autonomy was preserved through genuine self-expression, and competence was strengthened through sustained engagement and academic participation. These findings underscore that authenticity and motivation are profoundly relational and contextually situated processes rather than fixed individual traits.

Overall, the study demonstrates that authenticity serves as a core process in how adolescents experience and respond to witnessing bullying. When authenticity is supported through relational safety, students’ psychological needs are fulfilled, promoting self-esteem and autonomous motivation. When authenticity is constrained, need frustration contributed to avoidance, self-silencing, and disengagement. In this way, the middle-range grounded theory developed here extends self-determination theory (R. M. Ryan & Deci, 2017) and the literature on morality and bullying (e.g., Gutzwiller-Helfenfinger & Perren, 2022; Romera et al., 2019) by illuminating the lived and situated nature of autonomy, relatedness, and competence within the moral and emotional complexities of school life, thereby deepening our understanding of bystander behavior in bullying contexts.

## 5. Limitations

The study includes reliance on self-reported experiences, which may be influenced by recall bias or social desirability. Interview data may pose challenges in terms of ecological validity—that is, what students report about their own and their peers’ behaviors in an interview context may not fully correspond to what actually occurs in their everyday school life. Nevertheless, consistent with a constructivist grounded theory approach, the aim is not to produce an objective or literal representation, but rather an interpretative understanding of the phenomenon under study (Charmaz, 2014). The sample was limited to a small number of schools and may not represent all adolescent experiences. Thus, the small and non-probability sample constrains the transferability of the findings. However, rather than aiming for statistical generalization grounded in mathematical logic, qualitative research conceptualizes generalization as an interpretive process. As Larsson (2009) suggests, this involves generalization through recognition of patterns, whereby the reader—rather than the researcher—judges the relevance and transferability of the findings by recognizing similar patterns in other contexts. Nevertheless, future research could incorporate longitudinal or observational methods to capture how authenticity, self-esteem, and motivation evolve over time in bullying contexts. Additionally, the study primarily focused on in-person bullying, and cyberbullying was only minimally addressed; future research could further explore online bullying as a distinct context to understand how authenticity, self-esteem, and motivation are affected in digital settings.

## 6. Implications

Implications for practice include the importance of creating school environments where bystanders feel safe to act authentically. Interventions should extend beyond stopping bullying behaviors to foster supportive networks, encourage empathetic engagement, and validate students’ contributions to the school climate. Teachers and school staff can play a central role in facilitating protective relationships and reducing fear-driven avoidance, thereby supporting both psychological well-being and academic engagement. In addition, the findings point to several concrete areas for practical application. Teacher training programs can incorporate modules on recognizing subtle forms of social withdrawal, supporting students who adopt avoidance-based coping strategies, and facilitating constructive classroom climates that reduce fear of exposure. Schools may also strengthen peer support systems—by, for example, forming structured peer support systems and student-led support groups—to ensure that students have safe and reliable opportunities for social connection. Furthermore, intervention design can be enhanced by tailoring approaches to different motivational profiles: for students who cope through avoidance, interventions may focus on rebuilding safety and belonging, while for students who respond through performance-driven strategies, support may center on guiding them toward healthy academic engagement without overreliance on stress-related achievement. As such, participants’ insights into self-esteem and study motivation can inform interventions that move beyond simply stopping bullying behaviors, instead fostering environments where bystanders feel supported, valued, and equipped to contribute to a positive school climate.

## Data Availability

The data are not publicly available due to ethical restrictions.
